# Research on a Novel Exciting Method for a Sandwich Transducer Operating in Longitudinal-Bending Hybrid Modes

**DOI:** 10.3390/s17071510

**Published:** 2017-06-27

**Authors:** Yingxiang Liu, Qiangqiang Shen, Shengjun Shi, Jie Deng, Weishan Chen, Liang Wang

**Affiliations:** State Key Laboratory of Robotics and System, Harbin Institute of Technology, Harbin 150001, China; 1120810623@hit.edu.cn (Q.S.); sirssj@hit.edu.cn (S.S.); 16b908017@stu.hit.edu.cn (J.D.); cws@hit.edu.cn (W.C.); 17B308001@stu.hit.edu.cn (L.W.)

**Keywords:** piezoelectric transducer, longitudinal vibration, bending vibration, exciting method, electromechanical coupling factor

## Abstract

A novel exciting method for a sandwich type piezoelectric transducer operating in longitudinal-bending hybrid vibration modes is proposed and discussed, in which the piezoelectric elements for the excitations of the longitudinal and bending vibrations share the same axial location, but correspond to different partitions. Whole-piece type piezoelectric plates with three separated partitions are used, in which the center partitions generate the first longitudinal vibration, while the upper and lower partitions produce the second bending vibration. Detailed comparisons between the proposed exciting method and the traditional one were accomplished by finite element method (FEM) calculations, which were further verified by experiments. Compared with the traditional exciting method using independent longitudinal ceramics and bending ceramics, the proposed method achieves higher electromechanical coupling factors and larger vibration amplitudes, especially for the bending vibration mode. This novel exciting method for longitudinal-bending hybrid vibrations has not changed the structural dimensions of the sandwich transducer, but markedly improves the mechanical output ability, which makes it very helpful and meaningful in designing new piezoelectric actuators operated in longitudinal-bending hybrid vibration modes.

## 1. Introduction

Piezoelectric ultrasonic actuators (PUAs) have been seen as an important and preferential type of element for precision driving due to their merits of simple structure, small size, quick response, high resolution and a lack of electromagnetic radiation [1,2,3,4]. PUAs using sandwich transducers have attracted a lot of attention in recent years as the bolt-clamped sandwich structure not only improves output power and force, but also enhances working reliability [5,6,7,8,9,10]. Longitudinal-bending hybrid PUAs with the sandwich transducer structure have been proposed and discussed by some previous researchers, where the longitudinal vibration was used to produce the driving force, whereas the bending vibration served the role of overcoming preload.

Yun et al. proposed a novel PUA using a hybrid of the first longitudinal and second bending vibration modes of a sandwich transducer; their prototype achieved a no-load speed of 470 mm/s and a maximum force of 92 N [11]. Shi et al. proposed a longitudinal-bending hybrid PUA with exponential shape horns, which achieved a maximum speed and force of 920 mm/s and 45 N, respectively [12]. Zhang et al. designed a multi-degree of freedom (DOF) PUA under the operating principle of a longitudinal and bending vibrations hybrid, which was used to drive a robot finger joint [13]. Li et al. developed a novel 3-DOF PUA operated under longitudinal-bending hybrid vibration modes, in which a ball shaped rotor was rotated for 3-DOF motions [14]. Lee et al. tested an ultra-precision XY stage using longitudinal-bending hybrid bolt-clamped PUAs, in which the second longitudinal and eighth bending vibrations of a sandwich transducer were used to generate elliptical movement of the driving tip; their prototype achieved accuracy at the nanometer level [15].

All these works used sandwich transducers operating in longitudinal-bending hybrid modes, which meant that these two types of vibrations were generated simultaneously under the same frequency. It should be noted that the longitudinal and bending vibrations are excited by independent piezoelectric elements, which are named as longitudinal ceramics and bending ceramics, separately. Usually, the longitudinal vibration of a sandwich transducer is generated by whole-piece type piezoelectric ceramic plates polarized in the thickness direction, whereas the bending vibration needed half-piece type piezoelectric ceramic plates with reverse polarizations. The difference between the longitudinal and bending ceramics required them to be located at different positions of the transducer, which limited their axial location; that is to say, the longitudinal and bending modes could not reach the maximum electromechanical coupling efficiency synchronously due to this spatial limitation.

In this work, a novel exciting method for a sandwich transducer operating in longitudinal-bending hybrid modes is proposed and discussed. The longitudinal and bending ceramics are placed at the same axial location by dividing a whole-piece type piezoelectric ceramic plate into three independent partitions: the upper partition, the center partition and the lower partition. The center partition is the actuator for the longitudinal vibration, while the two outer partitions are used for the excitation of the bending mode. The finite element method (FEM) is used to verify the feasibility and superiority of the proposed exciting method. The electromechanical coupling factors and vibration amplitudes are calculated and compared with those of the traditional method. Two sandwich transducers using the new and traditional exciting methods respectively were fabricated and measured.

## 2. Structure and Operating Principle

**Figure 1:**
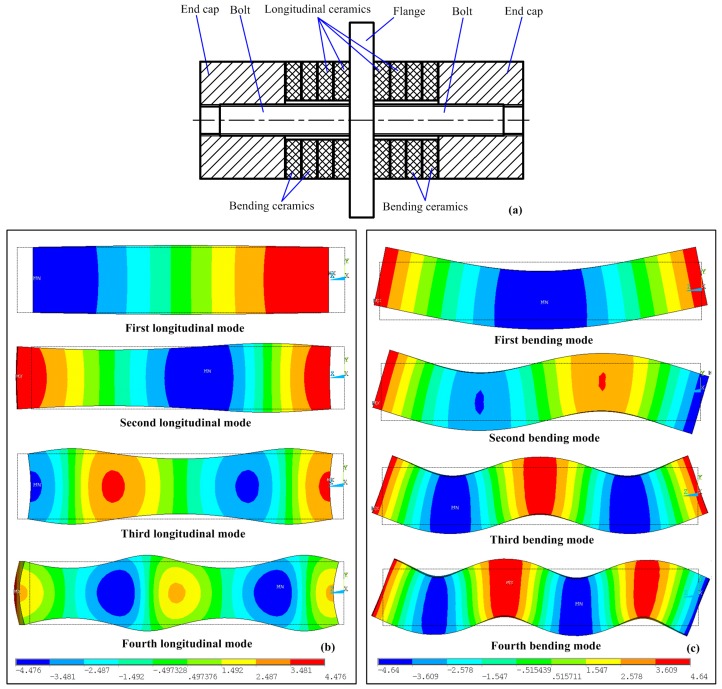
(**a**) The common structure of a sandwich transducer operating in longitudinal-bending hybrid modes; (**b**) longitudinal vibration modes of a beam with a square-shaped cross-section; (**c**) bending vibration modes of a beam with a square-shaped cross-section.

Figure 1a gives the basic structure of a bolt-clamped sandwich transducer operating in longitudinal-bending hybrid modes, which contains a flange with double head bolts, longitudinal piezoelectric ceramic plates, bending piezoelectric ceramic plates and two end caps; the piezoelectric ceramic plates are clamped between the flange and the end cap in sequence by the screw bolt, and the flange is also used for fixing. The vibration shapes of the first four order longitudinal and bending modes of a beam with a square-shaped cross-section are illustrated in Figure 1b,c, respectively. Usually, it is better to locate the flange near the wave nodes of both the longitudinal and bending modes as this setting will markedly reduce the effect of the mechanical fixing of the flange on the resonant vibration of the transducer. For this reason, longitudinal modes with odd number orders (first, third, etc.) and bending modes with even number orders (second, fourth, etc.) are the feasible options. It is common sense that the transducer can get a relatively higher vibration amplitude under a low order vibration mode; thus, the first longitudinal and second bending modes are most popular for the hybrid. Therefore, this work will use the first longitudinal-second bending hybrid modes to discuss the exciting method.

As an operating principle, the longitudinal vibration can produce axial simple harmonic vibrations on the two end tips, whereas the bending vibration will generate simple harmonic vibrations along the transverse direction. When these two simple harmonic vibrations have the same resonance frequency and a temporal shift of 90 degrees, elliptical movements will finally be formed, which can be used for driving if a runner is pressed into contact: the transverse displacement will overcome the preload and the axial displacement can push the runner linearly. Thus, the key problem in the design of a longitudinal-bending hybrid sandwich transducer is the match between the two vibration modes used, which means that their resonance frequencies should be tuned as closely as possible. This requirement will bring limitations on the structural dimensions as the match of frequencies can only be satisfied under certain sizes.

## 3. Exciting Method for the Longitudinal-Bending Hybrid Vibrations

**Figure 2:**
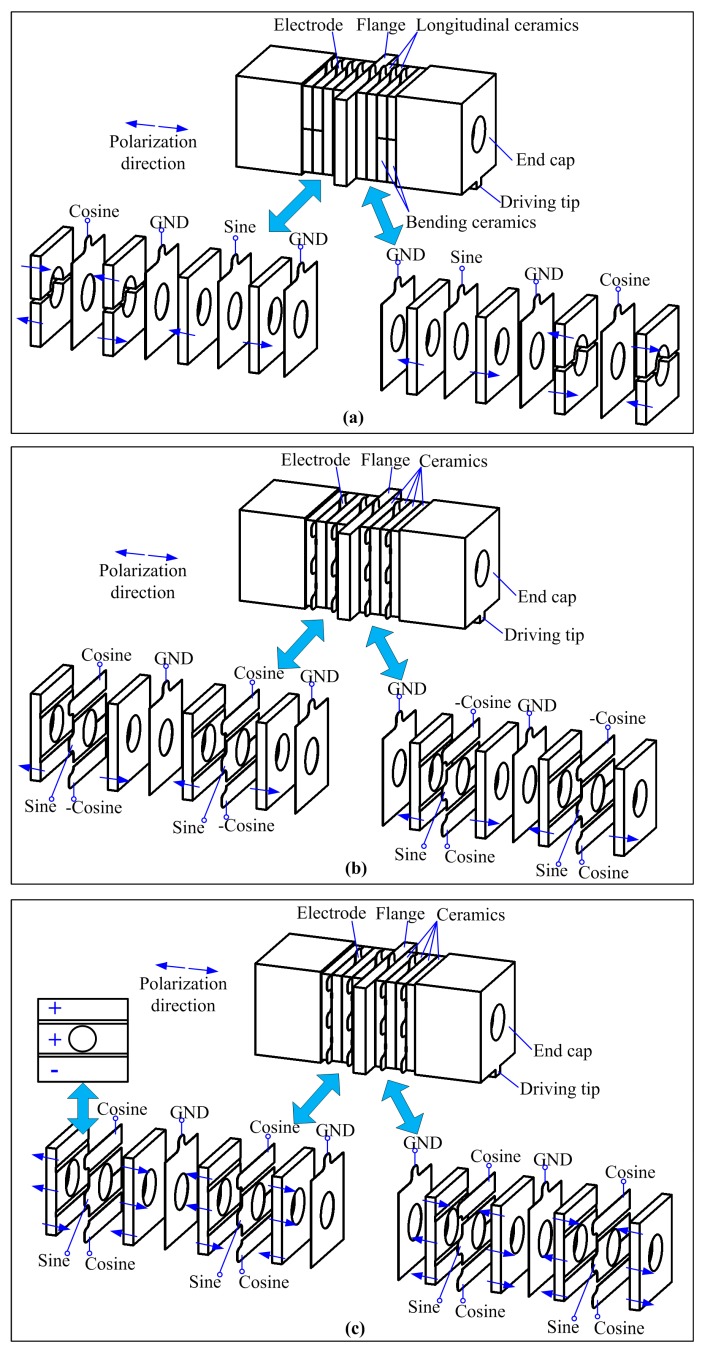
The arrangements of the piezoelectric ceramic plates and the applied exciting signals under different exciting methods: (**a**) the traditional exciting method for longitudinal-bending hybrid vibrations using separated longitudinal and bending ceramics; (**b**) the new exciting method for longitudinal-bending hybrid vibrations using whole-piece type ceramic plates with three uniform polarized partitions; (**c**) the new exciting method for longitudinal-bending hybrid vibrations using whole-piece type ceramic plates with three converse polarized partitions.

The aforementioned operating principle states that the longitudinal and bending vibration modes should be generated with a temporal shift of 90 degrees. The details of the traditional exciting method for the first longitudinal-second bending hybrid vibrations are illustrated in Figure 2a, in which the arrangements of the piezoelectric ceramic plates and the applied exciting signals are marked, and arrows are used to indicate the polarization directions. It can be clearly recognized that the piezoelectric elements are divided into two groups: longitudinal ceramics and bending ceramics, which are responsible for the excitation of the longitudinal and bending vibrations, respectively. The longitudinal ceramics are of the whole-piece type and have reverse polarizations with their neighbors, whereas the bending ceramics are of the half-piece type with reverse polarizations. Sine and cosine signals are applied to the two groups of piezoelectric elements, respectively, which favor the two vibrations with the desired temporal shift of 90 degrees. Under this exciting method, the longitudinal and bending ceramics are located at different axial positions of the transducer, and this may lead to structural limitations of the synchronous optimizations of the electromechanical coupling efficiencies of the two modes.

The new exciting method for a sandwich transducer operating in the first longitudinal-second bending hybrid modes proposed in this work is shown in Figure 2b,c. These two figures have the same idea in the excitation of the longitudinal-bending hybrid modes, in which the whole-piece type piezoelectric ceramic plates are divided into three independent partitions: the center partition is the actuator for the longitudinal vibration, while the two outer partitions are used for the excitation of the bending mode. In other words, the longitudinal and bending ceramics share the same axial location.

In Figure 2b, each piezoelectric ceramic plate has a uniform polarization direction on the three partitions, and the two neighboring ceramic plates have the reverse polarizations. Under these arrangements of the ceramic elements, three electrodes should be clamped between the neighboring ceramic plates, on which sine, cosine and -cosine signals are applied. The combination of the cosine and cosine signals can generate the second bending vibration as they may cause the anti-phase extension-shortening movements of the upper and lower partitions, respectively. The sine signal is used for the excitation of the first longitudinal vibration mode.

The exciting method shown in Figure 2b has a simple polarization process for the piezoelectric elements, but three phases of exciting signals are used. In comparison, the other exciting method shown in Figure 2c uses only two phases of exciting signals, but has a more complicated piezoelectric element polarizing: the three partitions of each piezoelectric ceramic plate have reverse polarizations, as shown by the arrows in Figure 2c; this change suggests that the second bending vibration mode can be excited by only applying a cosine signal. These two methods have not changed the structural dimensions of the transducer in comparison with the traditional method shown in Figure 2a. However, the exciting method shown in Figure 2c is more acceptable from the viewpoint of decreasing the complexity of the power supply since it only needs two phases of exciting signals. Thus, the exciting method shown in Figure 2c is selected for the following analysis and experiments.

## 4. Comparison between the Exciting Methods

The electromechanical coupling factors and the vibration amplitudes of the transducers under the traditional and the new exciting methods were calculated by FEM (ANSYS software) to illustrate the differences. Firstly, two FEM models were built separately according to the arrangements of the piezoelectric ceramic plates shown in Figure 2a,c, respectively. The structural dimensions of the transducer are: total length of 92 mm, ceramic plate of 35 mm × 35 mm × 4 mm, flange of 55 mm × 35 mm × 4 mm and end cap of 37 mm × 37 mm × 27.5 mm. The material of the flange is bronze (mass density *ρ* = 8300 kg/m^3^, Young modulus *E* = 1.07 × 10^11^ N/m^2^, Poisson ratio *σ* = 0.37); the material of the end cap is duralumin alloy (mass density *ρ* = 2810 kg/m^3^, Young modulus *E* = 7.2 × 10^10^ N/m^2^, Poisson ratio *σ* = 0.33) and the material of the ceramic plate is PZT-4, whose physical parameters are:

(1) $$d=\left[\begin{array}{cccccc} 0 & 0 & 0 & 0 & 5 & 0\\ 0 & 0 & 0 & 5 & 0 & 0\\ -1.6 & -1.6 & 3.3 & 0 & 0 & 0 \end{array}\right]\times 10^{-10}C/N$$

(2) $$c^{E}=\left[\begin{array}{cccccc} 15 & 8.4 & 6.8 & 0 & 0 & 0\\ 8.4 & 15 & 6.8 & 0 & 0 & 0\\ 6.8 & 6.8 & 12.9 & 0 & 0 & 0\\ 0 & 0 & 0 & 3.3 & 0 & 0\\ 0 & 0 & 0 & 0 & 2.8 & 0\\ 0 & 0 & 0 & 0 & 0 & 2.8 \end{array}\right]\times 10^{10}{N/m}^{2}$$

(3) $$\varepsilon^{T}=\left[\begin{array}{ccc} 8.1 & 0 & 0\\ 0 & 8.1 & 0\\ 0 & 0 & 6.7 \end{array}\right]\times 10^{-9}F/m$$

where *d*, *c^E^*, and *ε^T^* are the piezoelectric matrix, the stiffness matrix and the dielectric matrix, respectively.

**Figure 3:**
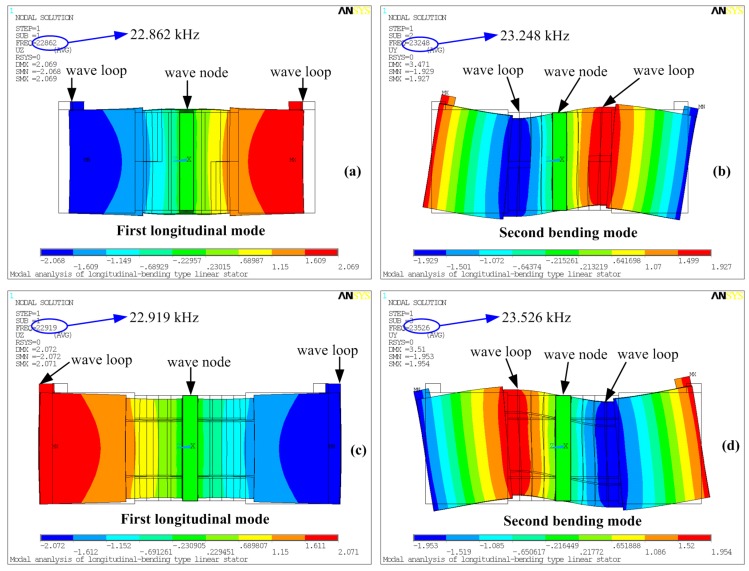
Vibration shapes and resonance frequencies of the transducer under different exciting methods: (**a**) first longitudinal mode under the traditional exciting method; (**b**) second bending mode under the traditional method; (**c**) first longitudinal mode under the new exciting method; (**d**) second bending mode under the new method.

Modal analyses were then developed based on the two models, from which the resonance frequencies of the first longitudinal and second bending modes were gained, as shown in Figure 3. It can be seen that the two resonance frequencies rise slightly when the new exciting method is applied, and the discrepancy between the two calculated longitudinal resonance frequencies is about 57 Hz, while this value is about 278 Hz for the bending mode. These differences are mainly caused by the separation of three partitions on one piezoelectric ceramic plate in the new exciting method. The width of the center partition used for the longitudinal mode is 19 mm, the widths of the upper and lower partitions for the bending mode are 7.5 mm, and there is also a region of isolation with a width of 0.5 mm between the neighboring partitions, which is set with no piezoelectric effect.

**Figure 4:**
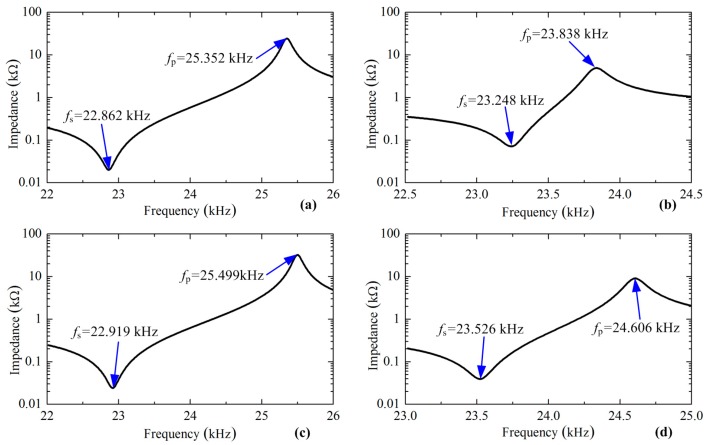
Input impedance characteristics of the transducer under different exciting methods: (**a**) first longitudinal mode under the traditional exciting method; (**b**) second bending mode under the traditional method; (**c**) first longitudinal mode under the new exciting method; (**d**) second bending mode under the new method.

From Figure 3a,b it can be clearly recognized that the longitudinal ceramics are located near the wave node of the longitudinal mode, whereas bending ceramics are placed at the wave loop of the bending mode; these arrangements of the piezoelectric plates comply with the classic design rule of sandwich transducers [11,12]. The new exciting method changes the locations of the piezoelectric plates: the longitudinal ceramics are placed slightly away from the wave node of the longitudinal mode, while the bending ceramics are set away from the wave loop of the bending mode. These changes may have effects on the two vibration modes. Thus, harmonic analyses were conducted to calculate the input impedance of the vibration modes under different exciting methods. The results are listed in Figure 4, from which the series resonance frequency *f_s_* and the parallel resonance frequency *f_p_* can be obtained by recognizing the points with minimum impedances and maximum impedances, respectively. The electromechanical coupling factor *k* is calculated by the following equation [16]:

(4) $$k=\sqrt{\frac{f_{p}^{2}-f_{s}^{2}}{f_{p}^{2}}}$$

Table 1 lists the calculated resonance frequencies and the electromechanical coupling factors, which gives a very clear comparison of the two exciting methods. It proves that the electromechanical coupling factor of the second bending mode has an increase of about 33% by using the new exciting method, while the first longitudinal mode shows a minute change. This phenomenon shows that we can get higher bending vibration amplitude under the same exciting signal. The electromechanical coupling factors of a previous transducer designed by Shi et al. are also listed in Table 1 to show the merits of the new exciting method; it should be noted that the parallel resonance frequencies are estimated values from the tested curve since they have not been provided directly.

**Figure 5:**
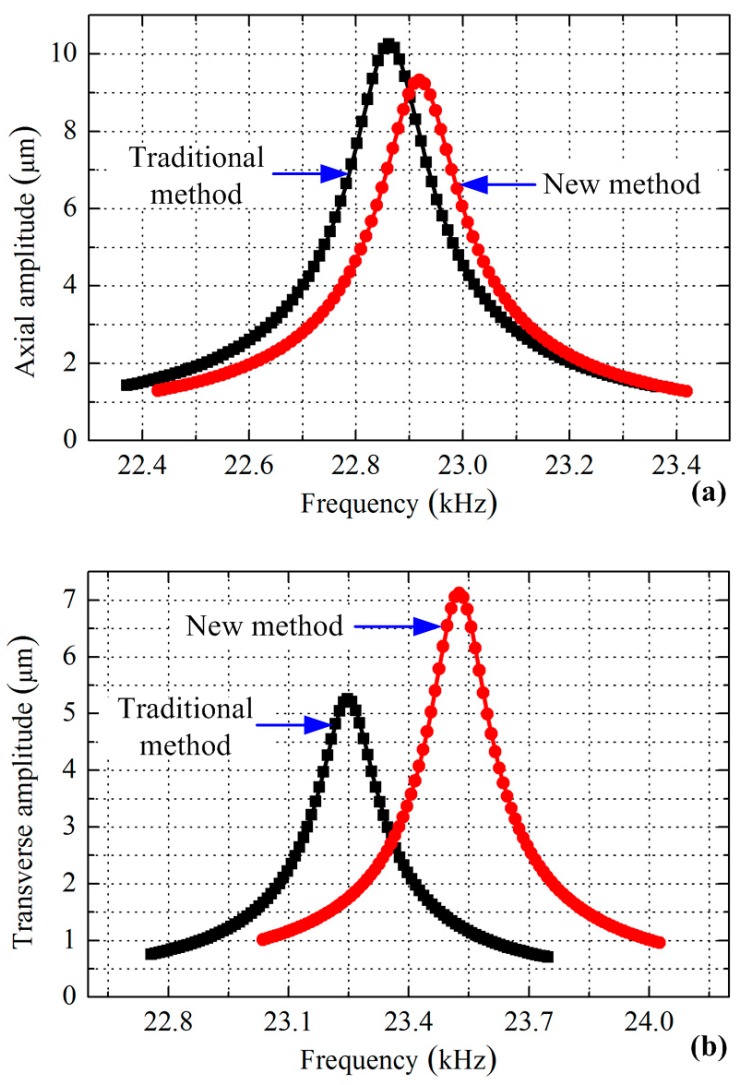
Comparison of the vibration amplitudes of the driving tip between different exciting methods by FEM: (**a**) axial amplitude under longitudinal vibration; (**b**) transverse amplitude under bending vibration.

Finally, the vibration amplitudes of the longitudinal and bending vibrations were calculated to further verify the merit of the new exciting method, during which voltage with an amplitude of 100 V and a damping factor of 0.003 were applied. Four calculations were developed in sequence for the two modes under the two exciting methods, respectively. The axial vibration amplitude of the driving tip was extracted for the longitudinal vibration, while the transverse amplitude was identified to represent the bending vibration. The gained curves of the amplitude versus the frequency are plotted in Figure 5.

Figure 5a indicates that the transducer can achieve the maximum axial amplitude of 10.2 µm at a frequency of 22.862 kHz by using the traditional exciting method, while these two values change to 9.3 µm and 22.919 kHz when adopting the new method. From the amplitude versus frequency curves shown in Figure 5b, it can be seen that the maximum transverse amplitude is increased from 5.3 µm to 7.1 µm by using the exciting method shown in Figure 2c.

These comparisons show that a great improvement is achieved in the bending vibration mode excitation, and a minute weakening occurs to the longitudinal mode. However, two other factors should be declared: the volumes of the piezoelectric elements used for the excitations and the optimal working frequencies, which are listed in Table 2. It can be seen that the decrease of the axial vibration amplitude is mainly caused by the reduction of the piezoelectric ceramic elements used. In addition, the bending vibration achieves an increase of about 34% on the maximum vibration amplitude under the new exciting method, less piezoelectric ceramic elements are used and the optimal frequency is higher than the traditional one—all these comparisons verify the outstanding advantages of the new method.

The improvements of the electromechanical coupling factors and the vibration amplitudes by the new exciting method can be explained by two reasons: Firstly, it changes the arrangements of the longitudinal and bending ceramics by using whole-piece type piezoelectric ceramic plates with three separated partitions, which favors their sharing the same axial location; moreover, the bending ceramics are placed away from the neutral plane of the bending vibration, which can also obviously improve the electromechanical coupling efficiency since the piezoelectric elements near the neutral plane contribute little to the generation of the bending mode.

## 5. Experiments

**Figure 6:**
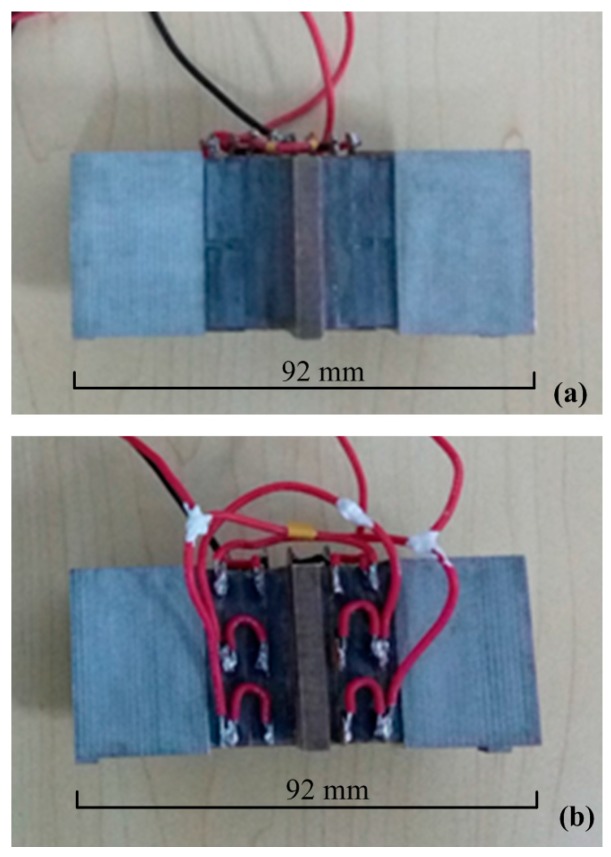
The prototypes of two transducers operating in longitudinal-bending hybrid modes under different exciting methods: (**a**) the one using the traditional exciting method; (**b**) the other one using the new exciting method.

**Figure 7:**
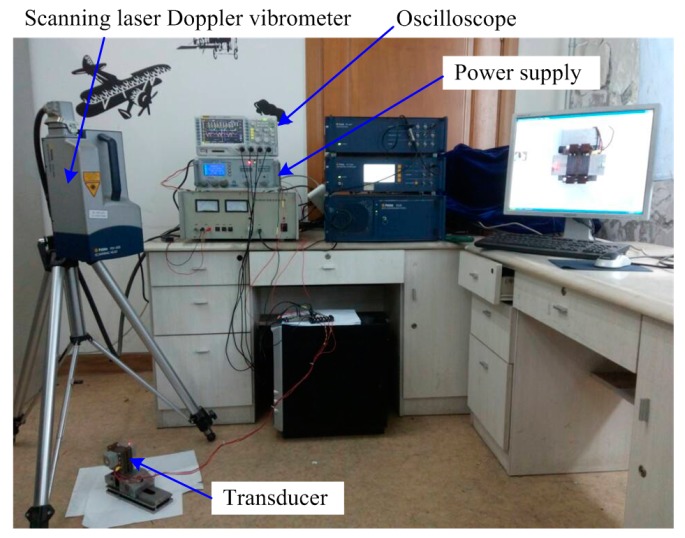
The experimental set-up for the measurement of the vibration amplitude.

**Figure 8:**
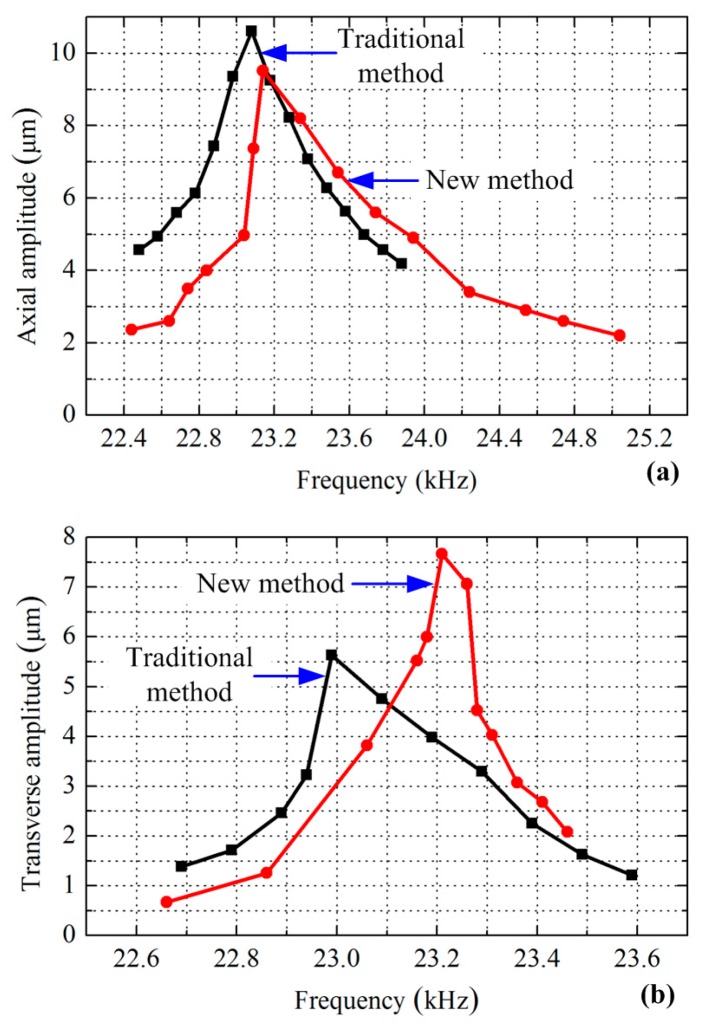
Comparison of the tested vibration amplitudes of the driving tip between different exciting methods: (**a**) axial amplitude under longitudinal vibration; (**b**) transverse amplitude under bending vibration.

Two sandwich transducers were fabricated using the traditional and the new exciting methods respectively, as shown in Figure 6. Whole-piece type ceramic plates with three converse polarized partitions were used to build the transducer using the new exciting method. The parameters of driving force and speed are usually used to evaluate the mechanical output ability of a sandwich ultrasonic motor, but it was hard to evaluate the longitudinal and bending vibrations separately using these two parameters since these two modes always have different resonance frequencies. The vibration amplitudes of the driving tips under different frequencies were more acceptable for the comparison of the exciting method. Therefore, the vibration amplitudes of the driving tips were measured by a scanning laser Doppler vibrometer (PSV-400-m2, Polytec, Waldbronn, Germany). The experiment set-up is shown in Figure 7, in which the transducer was excited by a power supply with a frequency tuning function, and the vibration of the driving tip was measured and recorded by the vibrometer. The tested vibration amplitudes of the driving tips under different exciting frequencies and a fixed voltage amplitude of 100 V are shown in Figure 8.

Figure 8a indicates that the first transducer reached the maximum axial amplitude of 10.6 µm at a frequency of 23.08 kHz by using the traditional exciting method, whereas the second transducer using the new exciting method achieved the maximum axial amplitude of 9.5 µm at a frequency of 23.14 kHz. Compared with the calculated results listed in Table 2, the longitudinal resonance frequencies show minute increases, and the maximum axial vibration amplitudes also show small increases. From Figure 8b, it can be clearly recognized that the maximum transverse amplitude was increased from 5.6 µm to 7.7 µm by using the exciting method, and the two bending resonance frequencies were 22.99 and 23.21 kHz, respectively. The two tested bending resonance frequencies show minute decreases in comparison with the one calculated by FEM, but the vibration amplitudes increased slightly. The discrepancies between the tested results and the calculated ones were mainly caused by the inaccuracy of the physical parameters of the materials, fabrication errors and assembly errors. However, the discrepancies between the resonance frequencies and the vibration amplitudes are very small, which can verify the results calculated by FEM and prove the advantages of the new exciting method effectively.

## 6. Conclusions

A new exciting method for the excitation of the longitudinal-bending hybrid vibrations of a sandwich piezoelectric transducer was proposed and simulated in this work. The structural dimensions, the total volume of piezoelectric elements and the exciting signals have not changed in comparison with the traditional exciting method. The changes concern the arrangements of the piezoelectric ceramic plates and the number of electrodes: each piezoelectric ceramic plate is divided into three separated partitions, which are linked with three individual electrodes. This method favors the piezoelectric elements for the longitudinal and bending vibrations sharing the same axial location. The electromechanical coupling factors and the vibration amplitudes were calculated and compared with those of the traditional method, which verified that the proposed exciting method obviously improved the bending vibration: the electromechanical coupling factor and the bending vibration amplitude increased by about 33 and 34%, respectively. The experiment results agreed well with those calculated by FEM. This new exciting method is not only available for the generation of first longitudinal-second bending hybrid vibration modes, but also suitable for the excitation of other combinations of longitudinal modes with odd number orders and bending modes with even number orders. Future work will focus on the practical use of this novel exciting method in designing a new linear piezoelectric ultrasonic actuator operating in longitudinal-bending hybrid modes.

## Figures and Tables

**Table 1:** The calculated resonance frequencies and electromechanical coupling factors under different exciting methods.

Exciting Method	Vibration Mode	*f*_s_ (kHz)	*f*_p_ (kHz)	*k* (%)
Traditional	Longitudinal	22.862	25.352	43.2
	Bending	23.248	23.838	22.1
New	Longitudinal	22.919	25.499	43.8
	Bending	23.526	24.606	29.3
Transducer by Shi et al. [12]	Longitudinal	29.38	31.37	35.1
	Bending	29.46	29.90	17.1

**Table 2:** The vibration analysis results under different exciting methods.

Exciting Method	Vibration Mode	Optimal Frequency (kHz)	Maximum Amplitude (µm)	Volumes of the Piezoelectric Elements Used (mm^3^)
Traditional	Longitudinal	22.862	10.2	17,137
	Bending	23.248	5.3	17,137
New	Longitudinal	22.919	9.3	16,354
	Bending	23.526	7.1	16,800
